# Association between lipid metabolism and cognitive function in patients with schizophrenia

**DOI:** 10.3389/fpsyt.2022.1013698

**Published:** 2022-11-24

**Authors:** Huamin Liu, Zhiwei Huang, Xiaochun Zhang, Yong He, Shanyuan Gu, Dan Mo, Shaoli Wang, Zelin Yuan, Yining Huang, Qi Zhong, Rui Zhou, Keyi Wu, Fei Zou, Xianbo Wu

**Affiliations:** ^1^Department of Epidemiology, Guangdong Provincial Key Laboratory of Tropical Disease Research, School of Public Health, Southern Medical University, Guangzhou, China; ^2^Baiyun Jingkang Hospital, Guangzhou, China; ^3^Department of Occupational Health and Medicine, Guangdong Provincial Key Laboratory of Tropical Disease Research, School of Public Health, Southern Medical University, Guangzhou, China

**Keywords:** lipids, lipoprotein cholesterols, apolipoproteins, cognitive impairment, schizophrenia, sphingolipids

## Abstract

**Background:**

The association between blood lipids and cognitive function in schizophrenia is still controversial. Thus, the present study aimed to verify the association between various lipid parameters and cognitive impairment in schizophrenic patients and potential lipid pathways.

**Methods:**

A total of 447 adult inpatients with schizophrenia were divided into cognitive normal and cognitive impairment groups based on the Mini-Mental State Examination with a cut-off of 26. The blood lipid parameters were defined as abnormal levels based on the guideline. The liquid chromatography-mass spectrometry method was used to preliminarily explore the potential lipid metabolism pathway associated with cognitive impairment.

**Results:**

There were 368 (82.3%) patients who had cognitive impairment. Herein, apolipoprotein B was positively associated with cognitive function in overall patients and age (≥45 and <45 years) and sex subgroups. After excluding patients with hypertension and diabetes, ApoB was still significantly associated with cognitive function in all the patients. The associations between other lipid parameters, including non-high-density lipoprotein cholesterol, low-density lipoprotein cholesterol, high-density lipoprotein cholesterol and triglyceride, and cognitive impairment were heterogeneous in age and sex subgroups. In contrast, total cholesterol and apolipoprotein A1 were not significantly associated with cognitive impairment. Metabolomics analysis showed that metabolic pathway mainly involved sphingolipid metabolism. Meanwhile, sphinganine and 3-dehydrosphinganine were positively correlated with lipid parameters and decreased in patients with cognitive impairment as compared to those with normal cognition.

**Conclusions:**

The present study suggests a positive association between lipids and cognitive function in schizophrenic patients and needs to be further verified by a prospective study.

## Introduction

Schizophrenia is a severe mental illness with poor recovery outcomes that is one of the top ten causes of long-term disability worldwide (1, 2). Cognitive impairment is a core feature of schizophrenia (3), linking to decreased adherence to treatment requirements, poor eating habits, and independent daily function in schizophrenia patients (4). It decreases the treatment effect of schizophrenia, induces other comorbidities, and declines the living quality of patients (5–7). Once cognitive impairment appears, it will be stable across the whole duration of schizophrenic patients (8). However, currently available antipsychotic therapy has little effect on cognitive function in patients with schizophrenia (9). Nonetheless, there is a subset of schizophrenic individuals who perform within the normal cognitive range (10, 11). Therefore, recognizing the risk factor for cognitive impairment in schizophrenia patients is essential and feasible for preventing cognitive impairment and advancing the rehabilitation of schizophrenic patients.

Patients with schizophrenia have a high risk experiencing of dyslipidemia (12, 13). In general, dyslipidemia is considered a risk factor for cognitive impairment, even a driving factor. However, current studies do not yield consistent results, such as an association between increased total cholesterol (TC) and better cognitive function, particularly verbal memory, was observed (14). Low-density lipoprotein cholesterol (LDL-C) and high-density lipoprotein cholesterol (HDL-C) included in TC have different influences on cognition based on the previous theory (15). Apolipoprotein B (ApoB) and apolipoprotein A1 (ApoA1) are the main structural proteins of LDL-C and HDL-C, respectively. However, a study found that ApoB and ApoA1 are both positively associated with immediate memory, language and attention in patients with schizophrenia (16). Moreover, a randomized controlled trial suggests that triglyceride (TG) might be an alternative energy substrate for the brain and have positive effects on cognitive function (17). We also found a similar result that non-high-density lipoprotein cholesterol (NHDL-C) is associated with better cognition in middle-aged and elderly people (18). Collectively, the above findings suggest the complexity of the association between blood lipids and cognitive function.

Metabolism is the final embodiment of the functional changes of upstream molecules (nucleic acids, proteins, etc.) in an organism, thus reflecting a series of pathophysiological events in the body under the influence of internal and external factors (19). Hence, metabolomics has an advantage in exploring precise pathways of diseases. Furthermore, antipsychotic treatment may induce metabolic disorders (20). However, which metabolic pathway is involved with cognitive impairment is still not well understood in schizophrenic patients.

Therefore, in the present study, a cross-sectional design was conducted in the present study to investigate the association between several lipid parameters, including TC, LDL-C, HDL-C, NHDL-C, TG, ApoA1, and ApoB, and cognitive impairment in hospitalized schizophrenic patients receiving regular antipsychotic treatment. In addition, non-targeted serum metabolomics analysis was used to validate whether or not lipid metabolism is involved with cognitive impairment in patients with schizophrenia. The present study will also provide new insight into the association between lipids, particularly the different types of lipid parameters and metabolic pathways, as well as cognitive impairments.

## Materials and methods

### Study population

Schizophrenic inpatients confined in Baiyun Jingkang Hospital, Guangzhou, China from 2020 to 2021 were recruited in this study. The inclusion criteria for patients were as follows: (1) patients who had been diagnosed with schizophrenia by experienced psychiatrists based on the International Classification of diseases-10 (ICD-10); (2) patients with stable psychotic symptoms for more than 2 weeks and able to finish the investigation and the cognitive function test; and (3) patients who were 18 years old or above. Meanwhile, the exclusion criteria for patients were as follows: (1) patients whose blood lipid measurements were missing; and (2) patients with history of cerebral trauma, stroke, myocardial infarction or dementia. A total of 489 inpatients were recruited in the current study. Based on the above exclusion criteria, 42 inpatients were excluded, and 447 inpatients were included in the analysis. The study abided by the Declaration of Helsinki principles. This current study was approved by the Medical Ethics Committee of Baiyun Jingkang Hospital. Written informed consent was obtained from each patient and family before performing any procedures related to this study.

### Blood lipids

The blood samples were collected after an overnight fast by medically trained nurses in the hospital. The full blood was extracted using an evacuated and promoting coagulating tube (Banbiao Medical Technology Co., Ltd., Guangzhou, Guangdong, China), and serum was obtained by centrifugation of 3000 rpm (DM0506 Centrifuge, Dalong Xingchuang Experimental Instruments Co., Ltd., Beijing, China). TC, LDL-C, HDL-C, TG, ApoA1, and ApoB were measured using an automatic Biochemical Analyzer (Mindray Medical International Co., Ltd., Shenzhen, Guangdong, China). NHDL-C was calculated by subtracting HDL-C from TC. Based on the 2016 Chinese guidelines for the management of dyslipidemia in adults, the high levels of TC, LDL-C, TG, NHDL-C, ApoA1, and ApoB are 5.2 mmol/L, 3.4 mmol/L, 1.7 mmol/L, 4.0 mmol/L, 1.9 mmol/L, 1.17 mmol/L, and above, respectively. Meanwhile, the low level of HDL-C is less than 1.0 mmol/L (21).

### Serum metabolomics test

A total of 15 patients with cognitive impairment were randomly selected, and then another 15 patients without cognitive impairment were matched based on age, sex, education, and use of antipsychotics such as clozapine, olanzapine, risperidone and quetiapine. Then, metabolomics tests were conducted on these 30 patients. The pre-treated serum was stored at −80°C. The frozen serum was thawed at room temperature before the metabolomics test. Then, 50 μL of the sample was transferred to an EP tube (Banbiao Medical Technology Co., Ltd., Guangzhou, Guangdong, China) and mixed with 200 μL of extract containing an isotopically labeled internal standard mixture (acetonitrile: methanol = 1:1, Banbiao Medical Technology Co., Ltd., Guangzhou, Guangdong, China). The samples were vortexed for 30 s, and sonicated for 10 min in an ice-water bath and incubated for 1 h at −40°C to precipitate the proteins. Subsequently, the sample was centrifuged at 12,000 rpm for 15 min at 4°C and then the supernatant was transferred to a fresh tube for analysis. Four quality control (QC) samples were then prepared by pooling equal amounts of the supernatant from all samples. In addition, blank samples interspersed throughout the experiment were used to evaluate cross-contamination among the samples.

A liquid chromatography-mass spectrometry (LC-MS) metabolomics test was performed using an ultra-high-performance liquid chromatography (UPLC) system (Vanquish, Thermo Fisher Scientific, Shanghai, China) with a UPLC-bridged ethyl hybrid amide column (2.1 mm × 100 mm, 1.7 μm) coupled with Q Exactive HFX MS (Orbitrap MS, Thermo Fisher Scientific, Shanghai, China) equipped with an electrospray ionization (ESI) source to obtain a comprehensive serum metabolic feature. The A phase of the mobile phase included 25 mmol/L of ammonium acetate and 25 mmol/L of ammonia hydroxide in water (pH 9.75), and the B phase was acetonitrile (Banbiao Medical Technology Co., Ltd., Guangzhou, Guangdong, China). The auto-sampler temperature was set at 4°C and the injection volume was 3 μL.

Q Exactive HFX MS can acquire tandem MS data in the information-dependent acquisition mode of the acquisition software (Xcalibur, Thermo Fisher Scientific, Shanghai, China). The acquisition software continuously evaluates the full-scan MS spectrum. In this study, the detailed parameters of the ESI source were as follows: sheath gas flow rate, 30 Arb; aux gas flow rate, 25 Arb; capillary temperature, 350°C; full MS resolution, 60,000; tandem MS resolution, 7,500; collision energy (CE), 10/30/60 in normalized CE mode; and spray voltage, 3.6 kV (positive) or −3.2 kV (negative).

A series of QC was performed in metabolite examination. First, instrument stability was demonstrated to be excellent *via* total ionization chromatography (Supplementary Figure 1). Data acquisition stability was also excellent based on the differences in the internal standard peaks amongst the QC samples (Supplementary Figure 2). There were no remarkable peaks observed for all internal standards in the blank samples, thus indicating that no cross-contamination occurred (Supplementary Figure 3).

The raw data were converted to mzXML format by using ProteoWizard and processed with an in-house program, which was developed using R 4.1.3 (R Foundation for Statistical Computing, Vienna, Austria) based on XCMS for peak detection, extraction, alignment and integration. Then, an in-house MS2 database (BiotreeDB) was applied for metabolite annotation with a cut-off of 0.3. The respective metabolic pathways of the different metabolites were annotated using the MetaboAnalyst.^1^

### Cognitive assessment

The global cognitive function of each patient was tested using the Mini-Mental State Examination (MMSE) by a well-trained psychiatrist within the psychiatric hospital. As a cognitive screening tool, the MMSE test is extensively applied for the early detection of cognitive impairment and it has been validated in schizophrenia (22, 23). The total score of MMSE is 30 and the cut-off point used for cognitive impairment screening was 26, which was considered to be good at discriminating cognitive impairments using MMSE in a previous scoping review (24). Thus, patients with MMSE scores of 26 and below were considered to have cognitive impairments.

### Covariates

Covariates were collected from the medical records of each inpatient and the course of schizophrenia was measured in years. Education level was categorized as elementary school or below, and middle school or above. Meanwhile, body mass index (BMI) was calculated based on the standard definition (weight/height^2^, kg/m^2^). The use of antipsychotics was from the prescription of the psychiatrist, and the actual usage of antipsychotics was corrected based on the test results of antipsychotics in the blood. Blood pressure was measured before the cognitive test, and hypertension was determined by recording a history of hypertension or systolic blood pressure ≥ 140 mmHg or diastolic pressure ≥ 90 mmHg or taking antihypertensive drugs. In addition, fasting blood glucose (FBG) and C-reactive protein (CRP) were measured using an automatic Biochemical Analyzer (Mindray Medical International Co., Ltd., Shenzhen, Guangdong, China). Moreover, diabetes was determined by recording the history of diabetes or FBG ≥ 7.0 mmol/L. Given the strict management of the hospital, the diet mode, physical activity level and sleeping habits of inpatients were similar, and they were not allowed to smoke or drink in the hospital. Hence, these factors were not selected as covariates.

### Statistical analysis

Given that continuous variables are non-normally distributed in the study, continuous variables were presented as the median and interquartile range (IQR). Gender and education level were presented as categorical variables. The Kruskal–Wallis test was employed for continuous variables test between cognitively impaired and cognitively normal groups, and the Chi-square test was used for categorical variables. A binary logistic regression model was applied to identify the associations of various blood lipid parameters with cognitive impairment. Model 1 was adjusted based on age and sex. Model 2 was adjusted based on age, sex, the course of schizophrenia, education, BMI, hypertension, FBG, CRP, and the use of clozapine, olanzapine, risperidone, and quetiapine. In addition, the subgroup analysis was used to explore the associations of blood lipids with cognitive function stratified by age (45 years) and sex. In sensitivity analysis, patients with history of hypertension and diabetes were excluded followed by the assessment of associations of blood lipids with cognitive impairment. The results of logistic regression were shown as odds ratios (ORs) and 95% confidence intervals (CIs). All statistical tests were two-sided, and the significance level was *P* < 0.05.

Meanwhile, the metabolomics data were separately processed in positive and negative ionization modes. Principal component analysis (PCA) and the correlation and differences in the internal standard responses of the QC samples suggested excellent data quality (Supplementary Figures 4–6 and Supplementary Table 1). The resultant data matrices were log-transformed and centralized then subjected to automated modeling analysis using the SIMCA software (V16.0.2, Sartorius Stedim Data Analytics AB, Umea, Sweden). Multivariate PCA was applied to determine the stability of the entire analysis process. Afterward, orthogonal projection to latent structures-discriminant analysis (OPLS-DA) was used to distinguish intergroup differences in the metabolic profile. The OPLS-DA model was validated by sevenfold cross-validation with R^2^ and Q^2^ and further validated by a permutation test with R^2^Y and Q^2^. The differences in peaks were tested by Wilcoxon rank-sum test and visualized in a volcano plot with *P* < 0.05 and variable importance in projection (VIP) > 1 in the first principal component in OPLS-DA. Moreover, a heat map was used to present the hierarchical clustering of the different metabolites that were successfully matched in the Human Metabolome Database. The data were processed by R version 4.1.3 software (R Foundation for Statistical Computing, Vienna, Austria).

## Results

### Characteristics of patients

Table 1 describes the demographic characteristics of the patients. A total of 82.3% (368/447) of patients with schizophrenia were diagnosed to have cognitive impairment. The median score of cognition among overall patients was 18 points (IQR: 10–24). Meanwhile, the score of the cognitive impairment group and the cognitively normal group were 15 points (IQR: 10–21) and 29 points (IQR: 28–30), respectively. The age of the patients ranged from 19 to 73 years, with a median age of 47 years (IQR: 39–56). The median age in the cognitive impairment group was 48 years (IQR: 39–57), and slightly higher than that in the cognitively normal group (46 years, IQR: 38–49, *P* < 0.001). Furthermore, the cognitively normal group had significantly better education, a lower rate of olanzapine use, and a higher rate of risperidone use than the cognitive impairment group. There were no differences observed in sex, course of schizophrenia, BMI, FBG, CRP, the prevalence of hypertension and diabetes, as well as the use of clozapine and quetiapine between the two groups.

**TABLE 1:** Characteristics of the patients with or without cognitive impairment.

Characteristics	Total	Cognitive normal	Cognitive impairment	*P*
*n*	447	79	368	
Cognitive score	18.0 [10.0, 24.0]	29.0 [28.0, 30.0]	15.0 [10.0, 21.0]	<0.001
Age (years)	47.0 [39.0, 56.0]	46.0 [38.0, 49.0]	48.0 [39.0, 57.0]	0.007
Males, *n* (%)	241 (53.9)	42 (53.2)	199 (54.1)	0.982
Course (years)	16.0 [8.0, 24.0]	14.0 [8.5, 20.0]	17.0 [8.0, 25.0]	0.087
Education, *n* (%)				
Illterate/Primary school	135 (30.2)	16 (20.3)	119 (32.3)	0.039
Above primary school	305 (68.2)	63 (79.7)	242 (65.8)	
TC (mmol/L)	4.6 [4.1, 5.3]	4.7 [4.2, 5.2]	4.6 [4.1, 5.3]	0.460
LDL-C (mmol/L)	2.7 [2.3, 3.2]	2.9 [2.5, 3.3]	2.7 [2.3, 3.2]	0.030
HDL-C (mmol/L)	1.1 [0.9, 1.3]	1.0 [0.8, 1.2]	1.1 [0.9, 1.3]	0.009
NHDL-C (mmol/L)	3.6 [3.0, 4.2]	3.7 [3.3, 4.2]	3.5 [2.9, 4.1]	0.082
TG (mmol/L)	1.3 [0.9, 1.9]	1.5 [1.1, 2.2]	1.3 [0.9, 1.9]	0.043
ApoAI (g/L)	1.4 [1.3, 1.6]	1.4 [1.3, 1.6]	1.4 [1.3, 1.6]	0.730
ApoB (g/L)	1.0 [0.8, 1.2]	1.1 [0.9, 1.2]	1.0 [0.8, 1.2]	0.001
FBG (mmol/L)	4.6 [4.3, 5.1]	4.5 [4.3, 5.0]	4.7 [4.3, 5.1]	0.089
CRP (mg/L)	3.0 [2.0, 6.0]	3.0 [2.0, 5.0]	3.0 [2.0, 6.0]	0.680
BMI (kg/m^2^)	22.7 [20.3, 25.3]	23.4 [20.8, 25.7]	22.6 [20.2, 25.1]	0.180
Hypertension, *n* (%)	74 (16.6)	10 (12.7)	64 (17.4)	0.390
Diabetes, *n* (%)	47 (10.5)	11 (13.9)	36 (9.8)	0.375
Clozapine, *n* (%)	58 (13.0)	12 (15.2)	46 (12.5)	0.645
Olanzapine, *n* (%)	115 (25.7)	10 (12.7)	105 (28.5)	0.005
Risperidone, *n* (%)	175 (39.1)	42 (53.2)	133 (36.1)	0.007
Quetiapine, *n* (%)	39 (8.7)	6 (7.6)	33 (9.0)	0.863

Quantitative variables: median [IQR]; qualitative variables: frequency (%). CN, cognitive normal; CI, cognitive impairment; TC, total cholesterol; LDL-C, low density lipoprotein cholesterol; HDL-C, high density lipoprotein cholesterol; NHDL-C, non-high density lipoprotein cholesterol; TG, triglyceride; ApoA1, apolipoprotein A1; ApoB, apolipoprotein B; FBG, fasting blood glucose; CRP, C-reaction protein; BMI, body mass index.

### Blood lipids between cognitive impairment and cognitively normal groups in schizophrenic patients

Patients with cognitive impairment had lower concentrations of LDL-C, TG, and ApoB, and higher concentrations of HDL-C than patients with normal cognition. There were no significant differences found in concentrations of TC and NHDL-C between the two groups (Table 1).

Significantly higher concentrations of TC, HDL-C, NHDL-C, and ApoA1 were observed in female versus male inpatients in the subgroup. As for age subgroups divided by the cut-off point of 45 years, TC, LDL-C, NHDL-C, and ApoB were significantly higher in patients aged 45 years or older (Supplementary Table 2).

### Association of blood lipids with cognitive impairment in patients with schizophrenia

**FIGURE 1:**
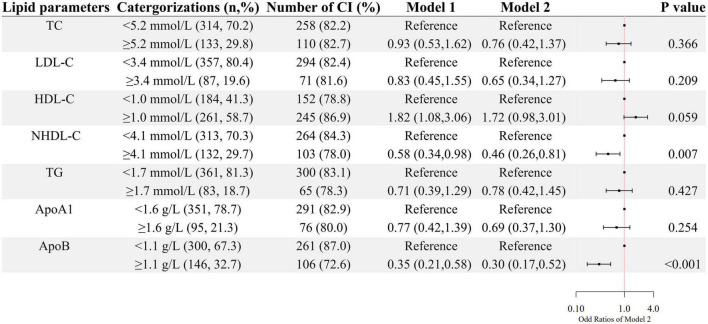
The association between lipid parameters and cognitive impairment. The ORs (95% CIs) of cognitive impairment were shown. Model 1 was adjusted by age and sex. Model 2 was adjusted by age, sex, the course of schizophrenia, education level, BMI, hypertension, FBG, CRP, and the use of clozapine, olanzapine, risperidone, and quetiapine.

A higher level of HDL-C was associated with higher odds of cognitive impairment after age and sex were adjusted, whereas, higher levels of NHDL-C and ApoB were associated with lower odds of cognitive impairment as shown in Figure 1. After further adjustment for education level, BMI, hypertension, FBG, and CRP, as well as the use of clozapine, olanzapine, risperidone and quetiapine, higher levels of NHDL-C, and ApoB were still associated with lower odds of cognitive impairment.

**FIGURE 2:**
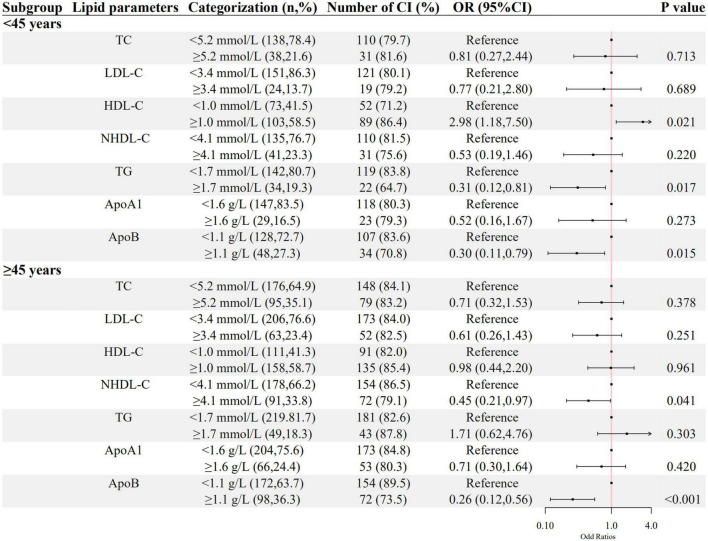
The association between lipid parameters and cognitive impairment stratified by 45 years. The ORs (95% CIs) of cognitive impairment were shown. The logistic regression model was adjusted by age, sex, the course of schizophrenia, education level, BMI, hypertension, FBG, CRP, and the use of clozapine, olanzapine, risperidone, and quetiapine.

**FIGURE 3:**
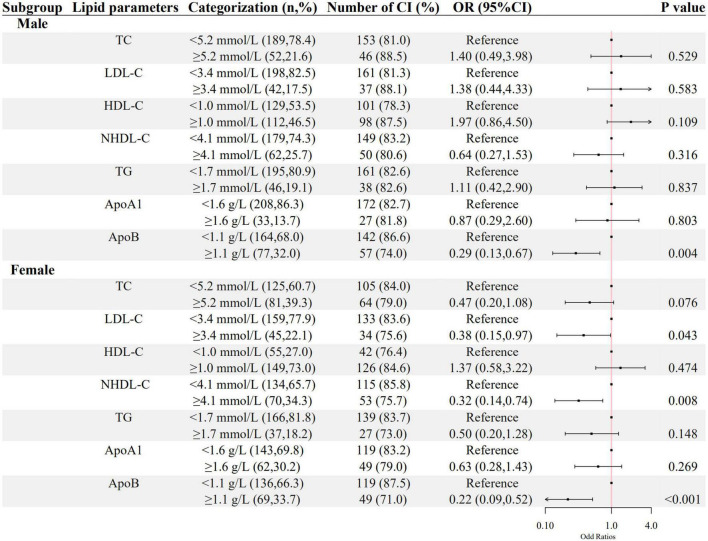
The association between lipid parameters and cognitive impairment stratified by sex. The ORs (95% CIs) of cognitive impairment were shown. The logistic regression model was adjusted by age, the course of schizophrenia, education level, BMI, hypertension, FBG, CRP, and the use of clozapine, olanzapine, risperidone, and quetiapine.

As shown in Figures 2, 3, a negative association of NHDL-C with cognitive impairment was observed in inpatients who were over 45 years or female in age and sex subgroups; ApoB was negatively associated with cognitive impairment in all subgroups; higher LDL-C was associated with lower odds of cognitive impairment in females, and higher TG was associated with lower odds of cognitive impairment in inpatients who were below 45 years old. HDL-C was positively associated with cognitive impairment only in patients below 45 years old.

**FIGURE 4:**
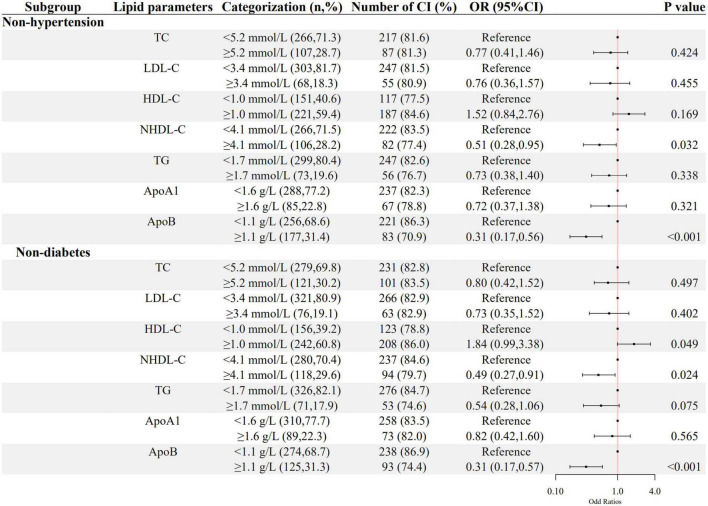
The association between lipid parameters and cognitive impairment in schizophrenic patients without hypertension and diabetes. The ORs (95% CIs) of cognitive impairment were shown. The logistic regression model was adjusted by age, sex, the course of schizophrenia, education level, BMI, hypertension, FBG, CRP, the use of clozapine, olanzapine, risperidone, and quetiapine.

The associations between ApoB, NHDL-C, and cognitive impairment in the sensitivity analysis were still significant in the inpatients without hypertension or diabetes, as shown in Figure 4.

### Metabolic characteristics of cognitive impairment and cognitively normal groups in patients with schizophrenia

The demographic characteristics among 15 patients with cognitive impairment and 15 cognitively normal were similar after they were matched (Supplementary Table 3).

The positive ionization mode was adopted because the OPLS-DA model in the negative ionization mode displayed low reliability (R^2^Y = 0.948, Q^2^ = –0.034). A total of 5,567 peaks were extracted in the positive ionization mode, and 3,933 peaks were used for actual analyses after filtration for single chromatographic peaks and data normalization (Supplementary Dataset 1).

**FIGURE 5:**
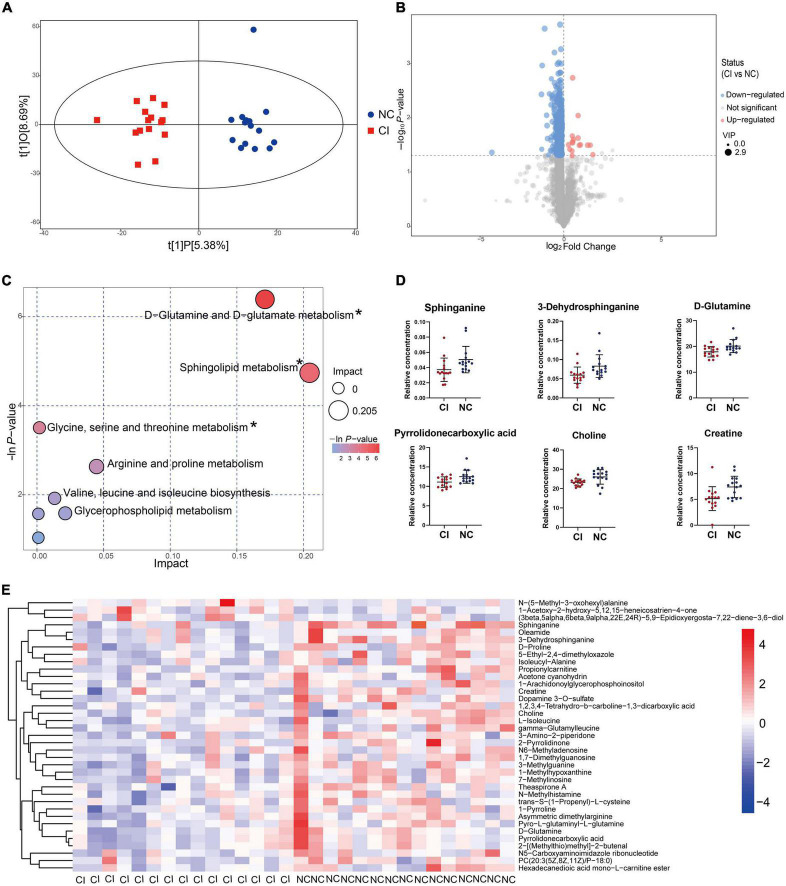
Metabolic difference between cognitive impairment and cognitively normal groups. **(A)** Score scatter plot of the OPLS-DA model. **(B)** Volcano plot for different metabolites. **(C)** Bubble plot showing metabolic pathways. **(D)** Key metabolites in the significantly enriched metabolic pathways. **(E)** Different metabolites previously identified. *P < 0.05.

The OPLS-DA model showed a remarkable separation of metabolism between schizophrenia with and without cognitive impairment (R^2^Y = 0.929, Q^2^ = 0.053, Figure 5A). The permutation test revealed that the OPLS-DA model did not produce overfitting (R^2^Y = 0.92, Q^2^ = –0.27, Supplementary Figure 7). There were 349 down-regulated and 16 up-regulated peaks found in cognitive impairment patients, with VIP > 1 in OPLS-DA and *P* < 0.05 (Figure 5B). However, only 37 metabolites (34 down-regulated and 3 up-regulated) were previously identified (see Figure 5E and Supplementary Table 4).

**FIGURE 6:**
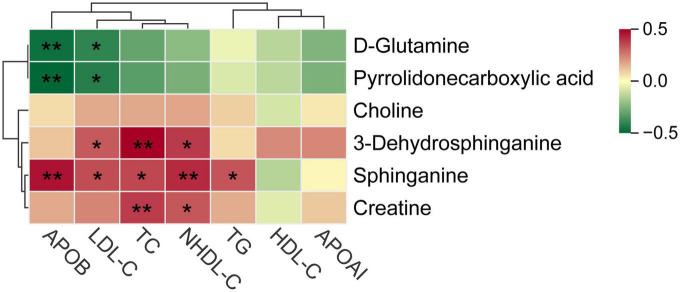
Spearman correlations between key metabolites and various lipid parameters. *P < 0.05, **P < 0.01.

The bubble plot shows the pathways that have evident enrichment. In lipid metabolism, sphingolipid metabolism was mainly involved by differential metabolites (*P* < 0.05, Figure 5C). The metabolites, including sphinganine and 3-dehydrosphinganine, decreased in the cognitive impairment group (Figure 5D and Supplementary Table 5), and positively correlated with TC, NHDL-C, LDL-C, and ApoB (Figure 6). In addition, D-glutamine and D-glutamate metabolism, glycine, serine and threonine metabolism were also involved, with significantly decreased metabolites of D-glutamine, pyrrolidonecarboxylic acid, choline and creatine in the cognitive impairment group.

## Discussion

The present study investigated the association between blood lipid metabolism and cognitive impairment in schizophrenic inpatients. The results of this study showed that a high level of ApoB was associated with better cognitive function in adult schizophrenic inpatients. NHDL-C was positively associated with cognitive function mainly in older (≥45 years) and female patients. Meanwhile, LDL-C was positively associated with cognitive function mainly in female patients. In addition, TG and HDL-C were positively and negatively associated with cognitive function in younger patients (<45 years), respectively. Metabolomics analysis showed that sphingolipid metabolism was the main lipid pathway that involved cognitive impairment.

Various previous studies suggest that the levels of distinct blood lipids are directly associated with cognitive function, however, the relationship between lipid profile and cognitive function has been inconclusive. Considering the role of unstable or abnormal levels of blood lipids in the body are associated with several undesirable outcomes, such as cardiovascular diseases, the hypothesis in most previous studies is that dyslipidemia might be a risk factor for cognitive impairment (25). Potential explanations of these results are that increased levels of LDL-C, TC, TG, and decreased HDL-C are associated with increased beta-amyloid (Aβ) plaques and hippocampal atrophy of the brain (25, 26). Cardiovascular disease and atherosclerosis induced by high levels of LDL-C, TC, and TG can also promote cognitive decline (27, 28).

However, we found in the current study that increased NHDL-C was associated with better cognitive function in schizophrenia. Similar relationships between cholesterol and cognitive function were found in previous studies. Such as, a prospective study found that higher TC is associated with better cognitive function in schizophrenia (29). Some studies also observed that higher TC and LDL-C levels are associated with better memory in community-dwelling elderly (30, 31). In our previous community cohort study, a longitudinal increase of NHDL-C is protective of cognition (18). Moreover, a study explored the association between cholesterol levels and cognitive function in patients with Parkinson’s disease and found that higher serum HDL-C is associated with poorer cognitive function (32), which coincides with our result in younger patients. Another longitudinal cohort study did not find any significant associations between HDL-C and cognitive impairment (33). Thus, in the present study, we guess the tendency of association between HDL-C and cognitive function might lead to an insignificant correlation between TC and cognitive impairment. Furthermore, for all the patients in the present study, it was NHDL-C rather than LDL-C that was associated with cognitive impairment, thereby indicating that very low-density lipoprotein-cholesterol (VLDL-C) and intermediate density lipoprotein-cholesterol (IDL-C) had potentially contributed to better cognition.

ApoB is mainly present in VLDL-C, IDL-C, and LDL-C, meanwhile, ApoA1 is mainly present in HDL-C (34). In the present study, we observed that ApoB was positively associated with cognitive function which was similar with another study in patients with chronic schizophrenia (16). In addition, increased ApoB was associated with decreased cerebrospinal fluid p-tau and t-tau in participants with subjective cognitive decline, which can explain why ApoB was associated with lower odds of cognitive impairment (35). However, the association between ApoA1 and cognitive impairment was not significant, which is consistent with the result of a randomized, double-blind, placebo-controlled clinical trial (33).

The above findings have several potential reasons. In humans, half of the cholesterol is biosynthesized in the liver (36) and a low cholesterol level may indicate poor liver function (37). Poorer liver function is associated with poorer cognitive function (38–40). A high level of cholesterol indicates a better nutritional status, which may benefit to cholesterol homeostasis in the brain (6, 41). Moreover, lipids are the main components of the brain with content next to adipose tissue and cholesterol responsible for the signal transduction of cell membranes (42). In this case, proper cholesterol level may be useful in maintaining normal brain function.

In age subgroups, we noticed that the positive association between NHDL-C and cognitive function was only significant in patients more than 45 years. Meanwhile, the negative association between HDL-C and cognitive impairment was only significant in patients less than 45 years. There is a study suggesting that a high HDL-C level (>1.55 mmol/L) is more strongly associated with cardiovascular mortality in adults less than 45 years than that in over 45 years (43), which may be the same for cognitive function. However, the potential reason for age heterogeneity is unclear.

Meanwhile, in sex subgroups, we found that LDL-C and NHDL-C were negatively associated with cognitive impairment in female patients. Only the ApoB was negatively associated with cognitive impairment in male patients. This finding was likely the result of the difference in endogenous sex hormones between males and females. In this study, most female patients were still menstruating (female median age: 48.00, IQR: 40.50–54.00). Cholesterol is the precursor of steroids. Thus, an increased level of cholesterol is followed by an increase in estrogen. The relatively high levels of endogenous estrogen are associated with better cognitive function (44–46). This synergetic increase of cholesterol and estrogens may explain the different sensitivity of blood lipids on cognitive function between males and females to a certain extent.

Atypical antipsychotic drugs (AADs) can induce metabolic syndrome, defined by components of abdominal obesity, glucose intolerance/hyperglycemia, hypertension, high TG and low HDL-C, all of which are correlated to cognitive function (47). Thus, we added AADs into the analysis in Model 2 to control the bias of antipsychotics. Interestingly, the associations between NHDL-C, ApoB, and cognitive function were still robust after adjusting the use of clozapine, olanzapine, risperidone, and quetiapine. Therefore, the associations of blood lipids with cognitive function in schizophrenics were likely independent of the effect of the above AADs. In fact, AADs have no direct effect on cognitive function. Clinical trials have indicated that there were no significant benefits of AADs to cognitive function (48).

Our metabolomics analysis confirmed that sphingolipid metabolism, a key lipid pathway, was involved in cognitive impairment. The results coincide with those of previous studies, in which sphingolipid metabolism was disordered, and sphinganine is decreased in the hippocampus of AD animals (49–51). Lipid rafts, the microdomain in the plasma membrane composed of cholesterol and sphingolipid, are essential to maintain the structural and functional integrity of cells and are responsible for cellular stabilization and signal transmission (52). Aβ can directly bind to cholesterol, and the toxicity of Aβ on various membrane organelles may be produced by disturbing the lipid raft domains (53). Hence, cholesterol and sphingolipid in serum may have a favorable effect on maintaining normal cognitive function. Sphinganine and 3-dehydrosphinganine hit the sphingolipid metabolic pathway and may be metabolic markers of cognitive impairment in patients with schizophrenia. However, the robust evidence should be further validated through a larger sample study.

A strength of this study is that the included patients had similar patterns of diet, exercise, and sleep mode, and they were also forbidden to smoke and take alcohol, which reduced the related confounding bias. There are some potential limitations in our study. First, a cross-sectional study cannot examine the causation. Nonetheless, this study provides a reference for clinical practice in the management of lipids in patients with schizophrenia. Second, we do not distinguish first-episode and recurrent schizophrenia. However, the metabolism status may be different between the two groups. Finally, our study focused on schizophrenic inpatients who took antipsychotics regularly, hence, it may not be generalized to patients who are not hospitalized.

## Conclusion

Increased NHDL-C and ApoB might be associated with better cognitive function in schizophrenic patients, meanwhile, HDL-C might be associated with poor cognitive function. The association of blood lipids with cognitive function was heterogeneous in age and sex stratification. In addition, sphingolipid metabolism may be a key metabolism pathway related to cognitive impairment, which should be noticed in patients with schizophrenia.

## Data availability statement

The raw data supporting the conclusions of this article will be made available by the authors, without undue reservation.

## Ethics statement

The studies involving human participants were reviewed and approved by the Medical Ethics Committee of Baiyun Jingkang Hospital. The patients/participants provided their written informed consent to participate in this study.

## Author contributions

HL and ZH wrote the article. XZ and YHe recruited the inpatients. SG provided the clinical guidance. DM organized the cognitive tests. SW was in charge of the blood sample tests. HL, ZH, ZY, YHu, QZ, RZ, and KW collected and collated the data. FZ and XW contributed to the study concept and design and reviewed the article. All authors contributed to the article and approved the submitted version.

## Footnotes

1
http://www.metaboanalyst.ca/
