# Supplementary material for: Association between lipid metabolism and cognitive function in patients with schizophrenia
Source: Front Psychiatry. 2022 Nov 24;13:1013698. doi: 10.3389/fpsyt.2022.1013698 (PMC9729695; doi:10.3389/fpsyt.2022.1013698)
Supplement: Supplementary file 1 [file Data_Sheet_1.docx]

**Supplementary results**


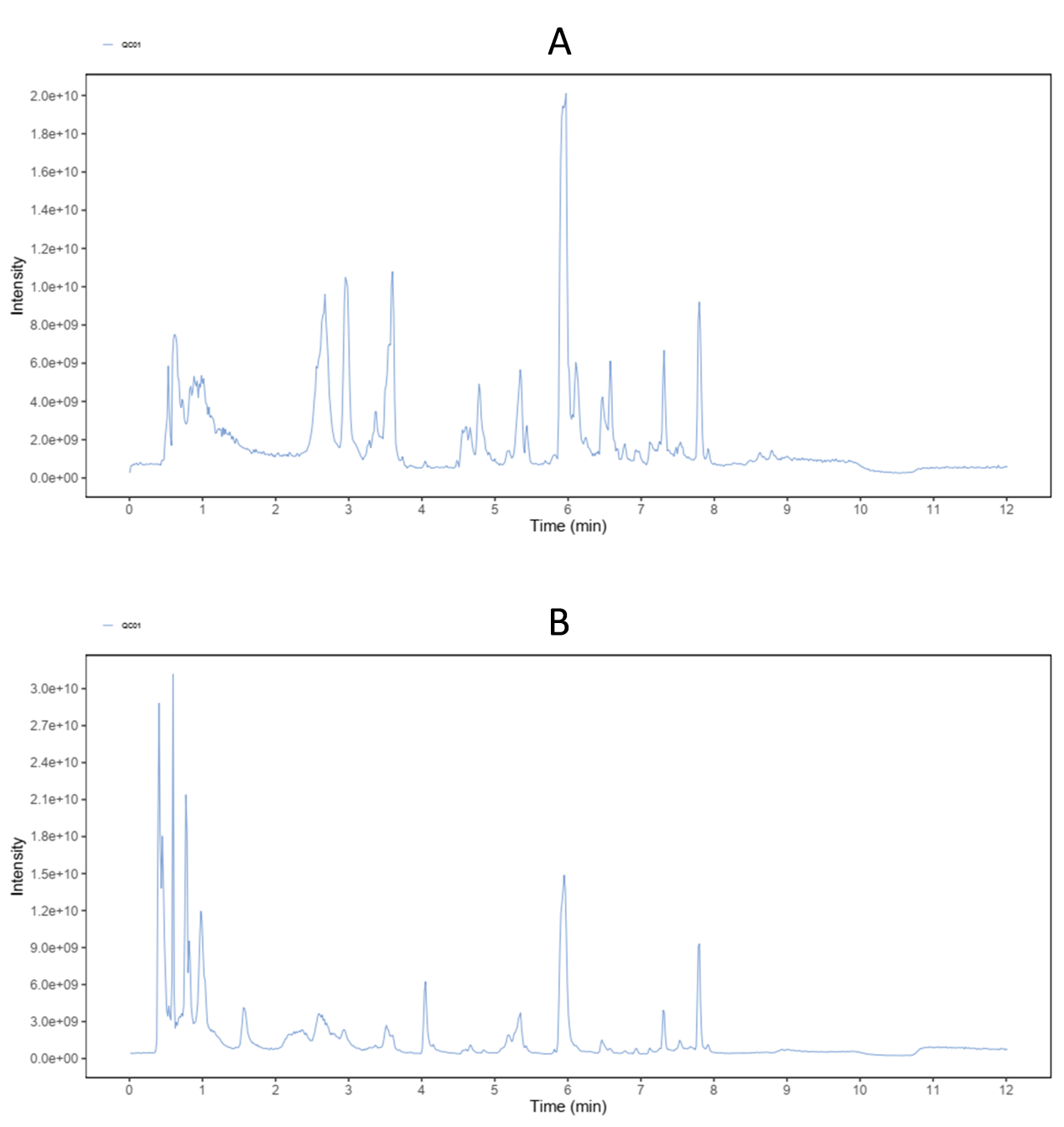


**Supplemental Figure 1** Total ion chromatogram (TIC) in positive ionization mode (A) and negative ionization mode (B)


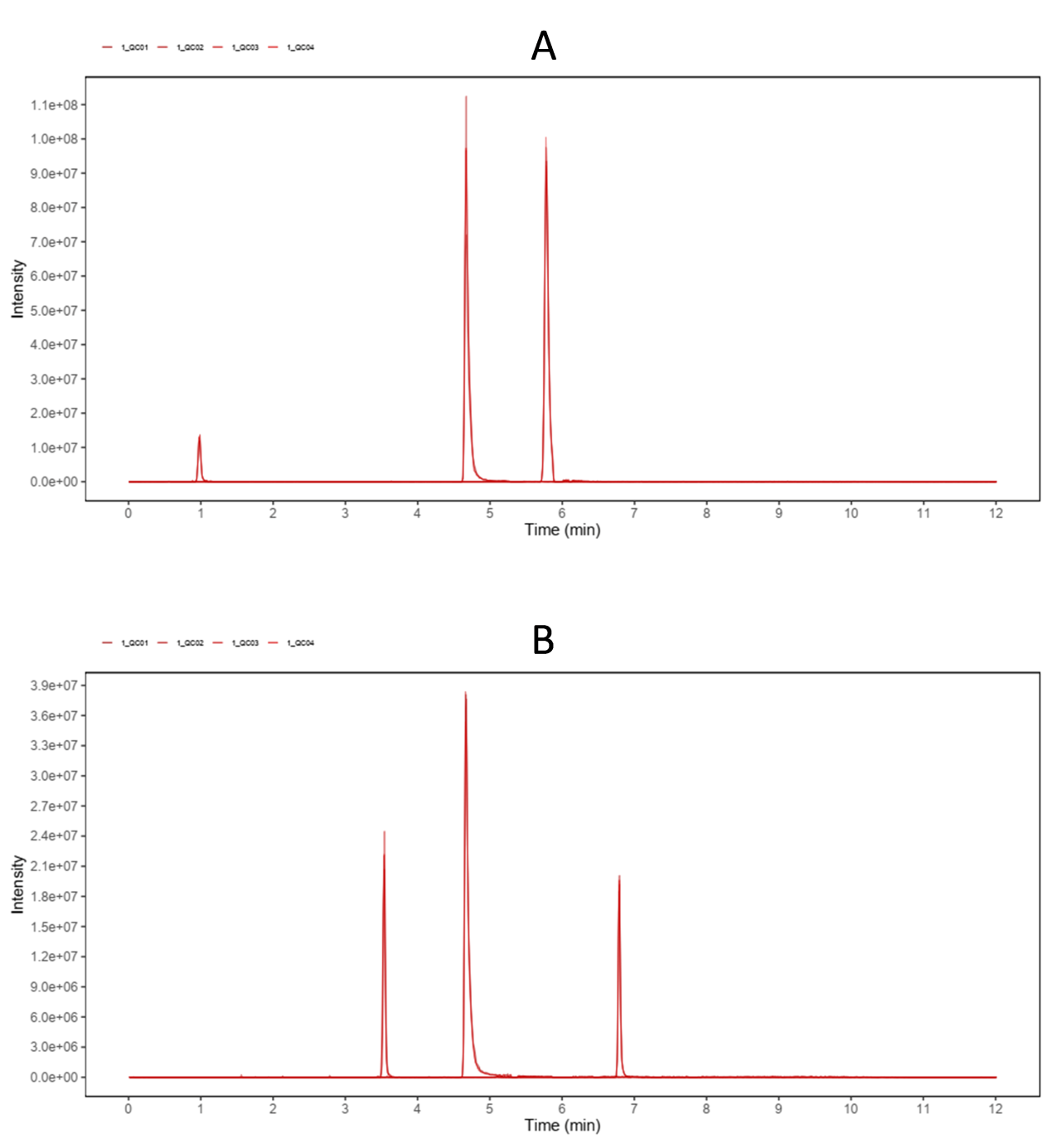


**Supplemental Figure 2** Extracted ion chromatogram (EIC) of internal standard among QC samples in positive ionization mode (A) and negative ionization mode (B)


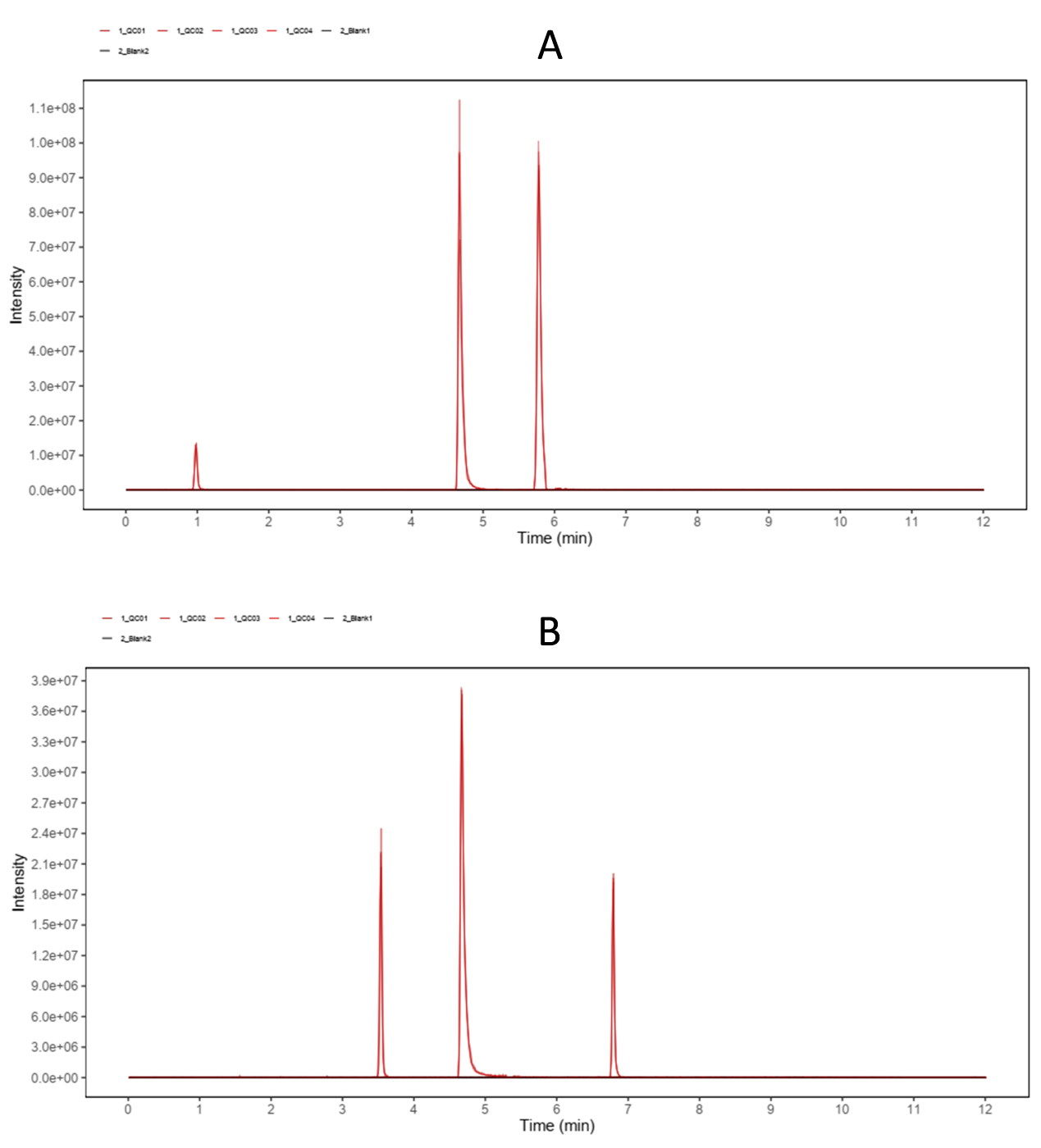


**Supplemental Figure 3** Extracted ion chromatogram (EIC) of internal standard among blank samples in positive ionization mode (A) and negative ionization mode (B)


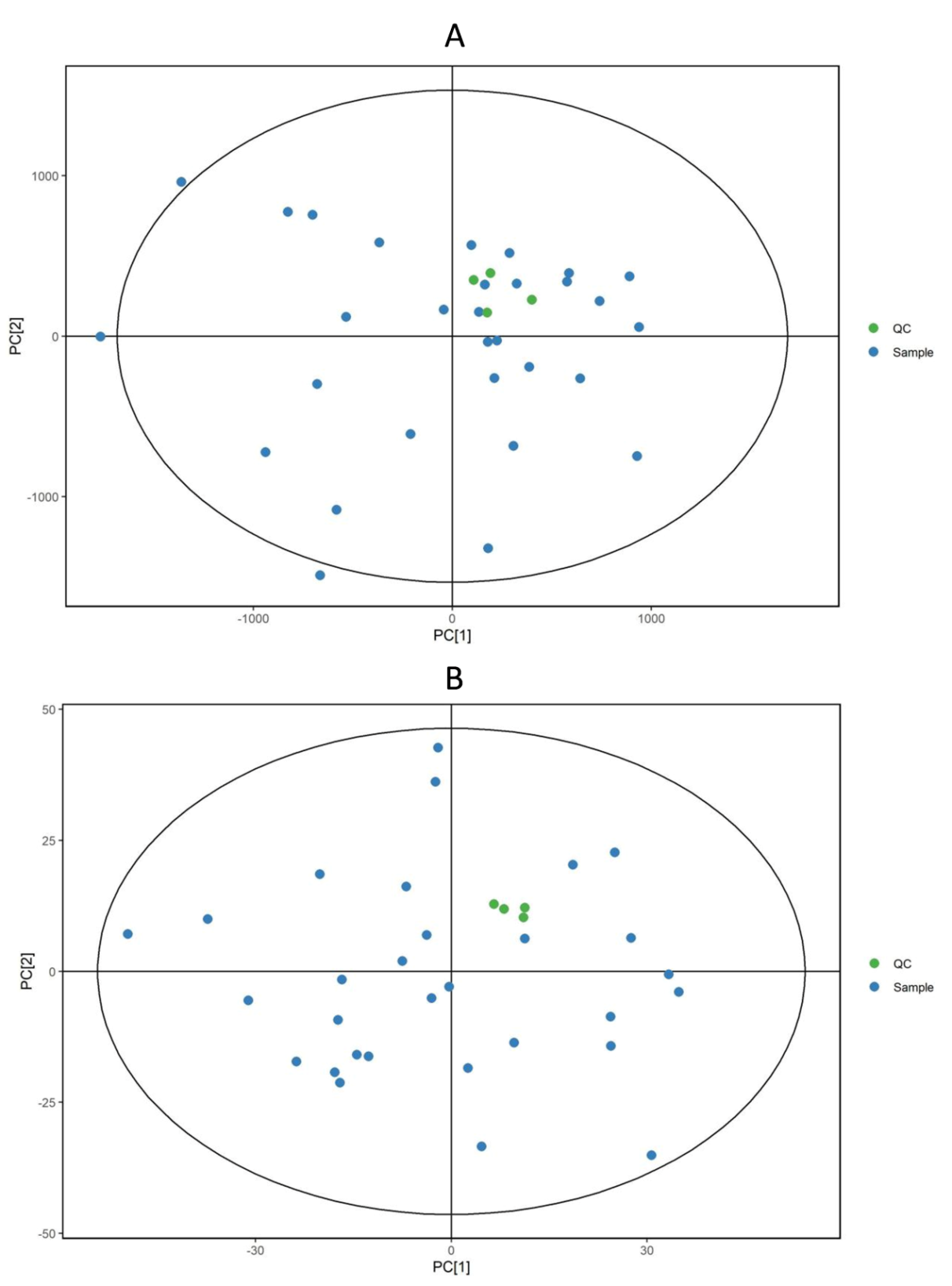


**Supplemental Figure 4** Principal component analysis (PCA) score of QC samples (green dot) and test sample (blue dot)


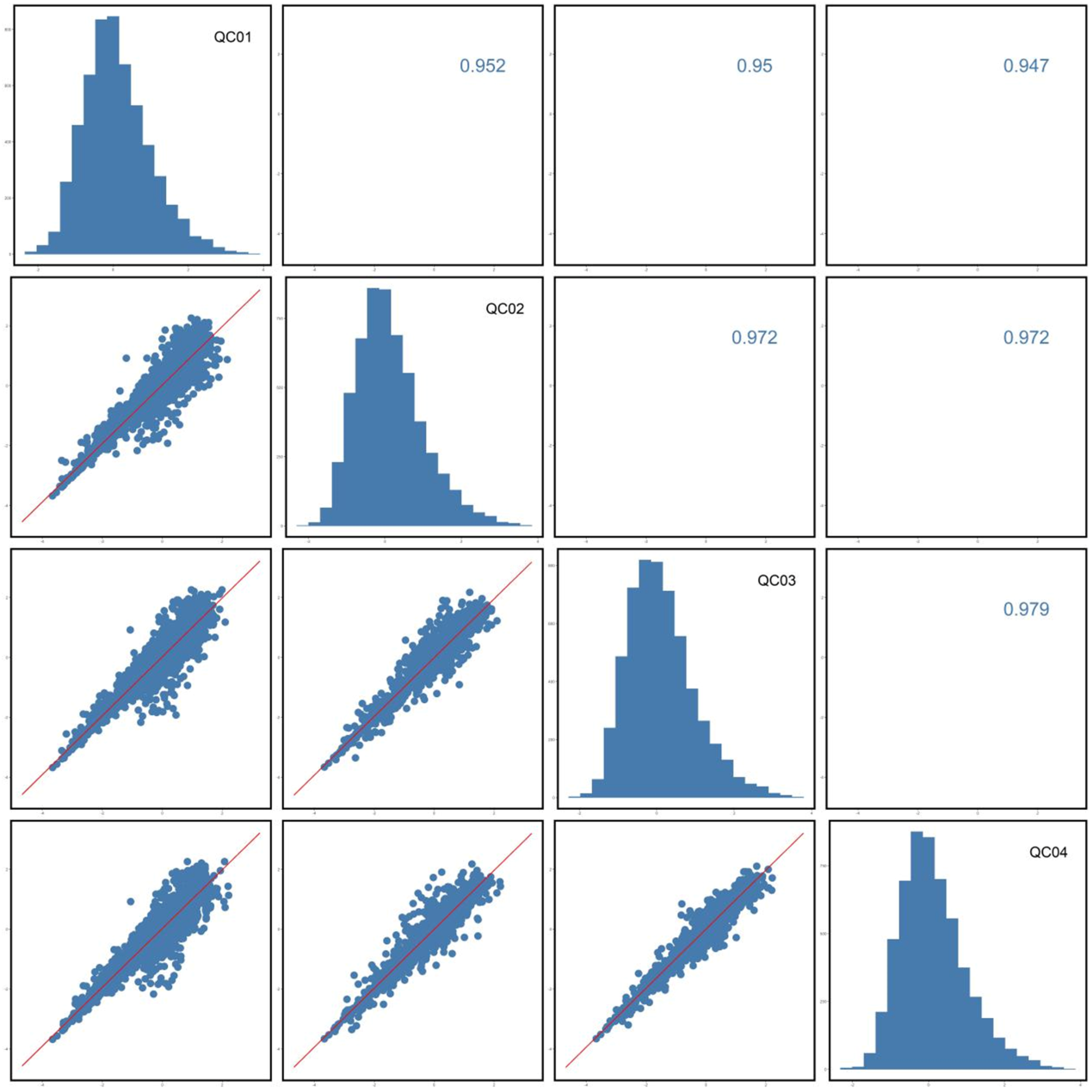


**Supplemental Figure 5** Correlations among QC samples in positive ionization mode


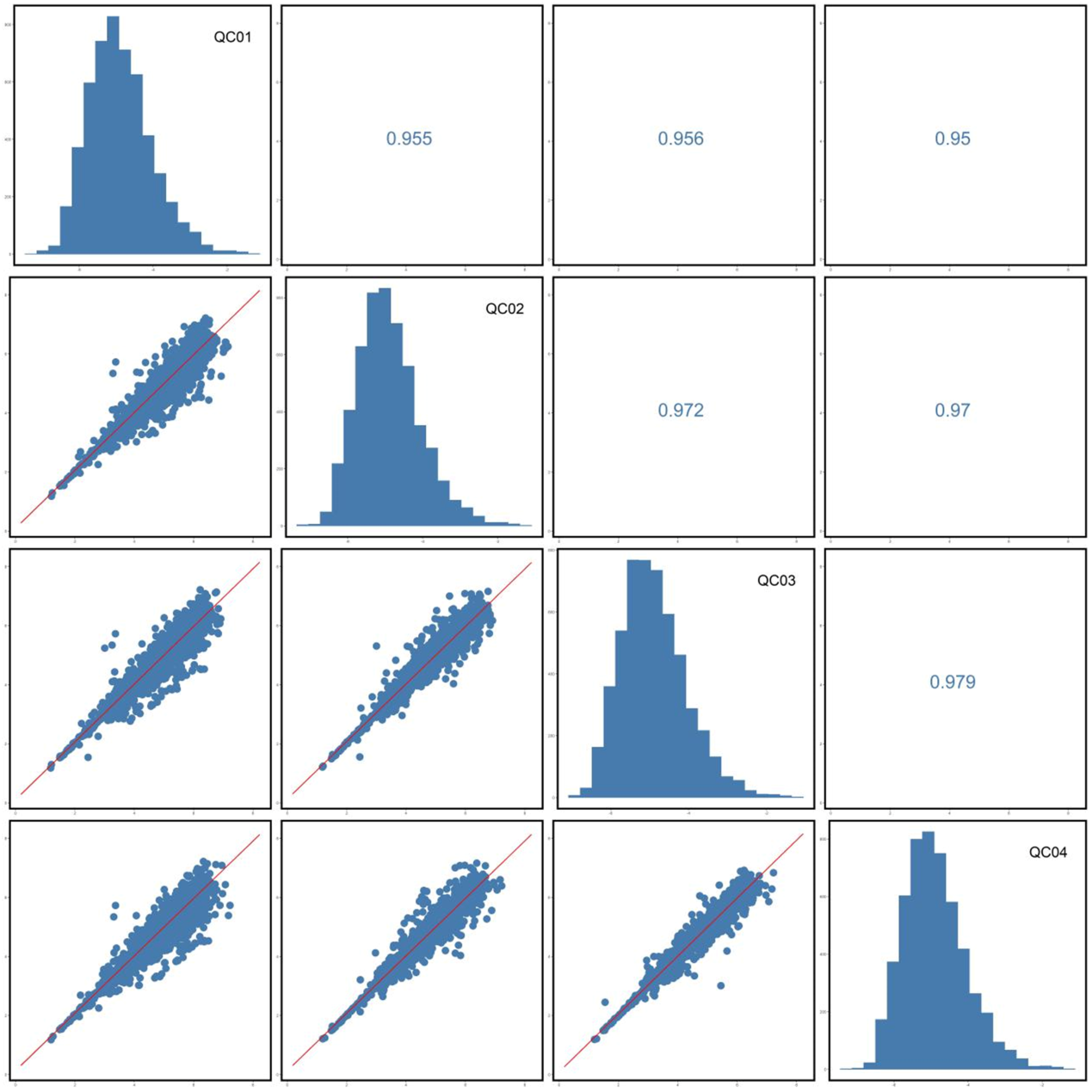


**Supplemental Figure 6** Correlations among QC samples in negative ionization mode

**Supplemental Table 1** Response stability of internal standard

| Internal standard | Positive ionization mode | | | | Negative ionization mode | | |
| --- | --- | --- | --- | --- | --- | --- | --- |
|  | rt (s) | m/z | RSD (%) | rt (s) | | m/z | RSD (%) |
| IS1 | 280.9110 | 135.1204 | 10.87 | 212.2045 | | 183.0818 | 1.96 |
| IS2 | 347.2325 | 85.1325 | 3.43 | 280.7280 | | 133.1056 | 4.56 |
| IS3 | 59.0695 | 127.0801 | 6.11 | 407.6940 | | 121.0438 | 2.59 |

rt, retention time (s); m/z, mass to charge ratio; RSD, relative standard deviation


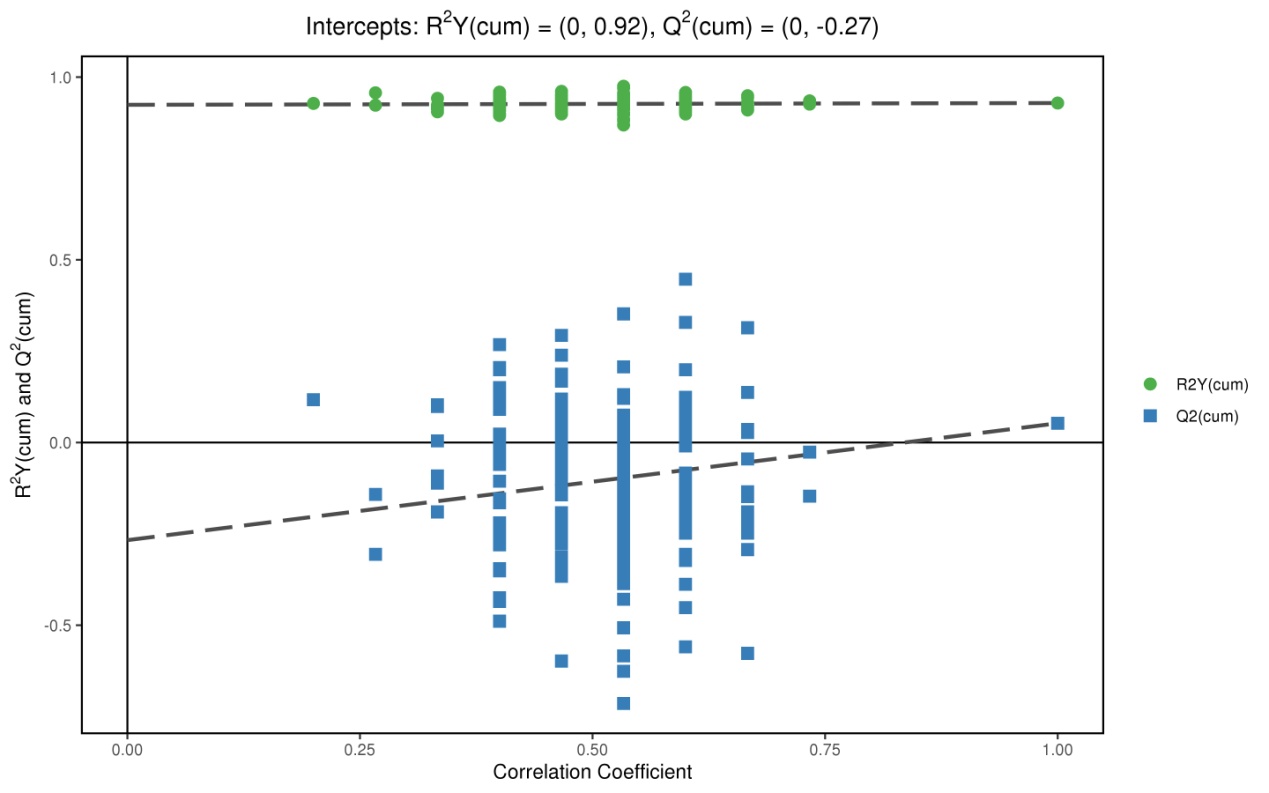


**Supplemental Figure 7** Permutation test of OPLS-DA model

**Supplementary Table 2** Characteristics of the patients stratified by age and sex

| Characteristics | <45 years | ≥45 years | *P* | Male | Female | *P* |
| --- | --- | --- | --- | --- | --- | --- |
| n | 178 | 299 |  | 262 | 215 |  |
| Cognitive score | 20.00 [14.00, 25.75] | 14.00 [9.00, 23.00] | <0.001 | 17.00 [10.00, 24.00] | 18.00 [10.00, 24.00] | 0.471 |
| Age (years) | 37.00 [31.00, 41.00] | 54.00 [49.00, 60.00] | <0.001 | 49.00 [39.00, 58.75] | 48.00 [40.50, 54.00] | 0.171 |
| Sex (Male) | 101 (56.7) | 161 (53.8) | 0.603 | - | - | - |
| Course (years) | 11.00 [6.00, 16.00] | 20.00 [11.00, 30.00] | <0.001 | 19.00 [10.00, 26.00] | 14.00 [7.25, 21.00] | 0.003 |
| Education |  |  |  |  |  |  |
| Primary school/Illterate | 47 (26.7) | 88 (32.5) | 0.296 | 86 (35.7) | 49 (23.8) | 0.022 |
| Above primary school | 125 (71.0) | 180 (66.4) |  | 151 (62.7) | 154 (74.8) |  |
| TC (mmol/L) | 4.50 [4.00, 5.10] | 4.70 [4.20, 5.40] | 0.003 | 4.50 [4.00, 5.00] | 4.90 [4.20, 5.55] | <0.001 |
| LDL-C (mmol/L) | 2.68 [2.28, 3.11] | 2.78 [2.31, 3.35] | 0.018 | 2.70 [2.31, 3.19] | 2.78 [2.31, 3.34] | 0.132 |
| HDL-C (mmol/L) | 1.04 [0.89, 1.22] | 1.06 [0.91, 1.31] | 0.155 | 0.98 [0.86, 1.18] | 1.15 [0.98, 1.40] | <0.001 |
| NHDL-C (mmol/L) | 3.46 [2.92, 3.98] | 3.60 [3.01, 4.30] | 0.013 | 3.46 [2.92, 4.12] | 3.64 [3.07, 4.30] | 0.036 |
| TG (mmol/L) | 1.40 [1.00, 1.90] | 1.30 [0.90, 1.90] | 0.528 | 1.30 [1.00, 1.90] | 1.30 [0.90, 1.90] | 0.537 |
| ApoAI (g/L) | 1.41 [1.31, 1.53] | 1.45 [1.32, 1.57] | 0.115 | 1.41 [1.30, 1.52] | 1.46 [1.35, 1.65] | <0.001 |
| ApoB (g/L) | 0.96 [0.78, 1.13] | 0.98 [0.82, 1.19] | 0.038 | 0.97 [0.80, 1.15] | 0.97 [0.79, 1.20] | 0.967 |
| FBG (mmol/L) | 4.47 [4.23, 4.94] | 4.76 [4.35, 5.26] | <0.001 | 4.56 [4.27, 5.04] | 4.79 [4.38, 5.19] | 0.004 |
| CRP (mg/L) | 3.00 [2.00, 6.00] | 3.00 [2.00, 7.00] | 0.034 | 3.00 [2.00, 6.00] | 3.00 [2.00, 7.00] | 0.439 |
| BMI (kg/m^2^) | 23.38 [21.01, 25.59] | 22.41 [19.99, 24.58] | 0.009 | 23.28 [20.20, 25.57] | 22.19 [20.35, 24.40] | 0.114 |
| Hypertension (Yes) | 7 (4.0) | 67 (24.7) | <0.001 | 47 (19.5) | 27 (13.1) | 0.092 |
| Diabetes (Yes) | 8 (4.5) | 39 (14.4) | 0.002 | 30 (12.4) | 17 (8.3) | 0.198 |
| Clozapine (Yes) | 22 (12.5) | 36 (13.3) | 0.923 | 35 (14.5) | 23 (11.2) | 0.362 |
| Olanzapine (Yes) | 49 (27.8) | 66 (24.4) | 0.476 | 62 (25.7) | 53 (25.7) | 1 |
| Risperidone (Yes) | 71 (40.3) | 104 (38.4) | 0.752 | 98 (40.7) | 77 (37.4) | 0.54 |
| Quetiapine (Yes) | 13 (7.4) | 26 (9.6) | 0.524 | 18 (7.5) | 21 (10.2) | 0.396 |
| Quantitative variables: median [IQR]; qualitative variables: frequency (%)  Abbreviations: CN, cognitive normal; CI, cognitive impairment; TC, total cholesterol; LDL-C, low density lipoprotein cholesterol; HDL-C, high density lipoprotein cholesterol; NHDL-C, non-high density lipoprotein cholesterol; TG, triglyceride; ApoA1, apolipoprotein A1; ApoB, apolipoprotein B; FBG, fasting blood glucose ; CRP, C-reaction protein; BMI, body mass index. | | | | | | |

**Supplementary Table 3** Characteristics of the patients participating in metabolomics analysis

| Characteristics | Total | Cognitive normal | Cognitive impairment | *P* |
| --- | --- | --- | --- | --- |
| n | 30 | 15 | 15 |  |
| Cognitive score | 27.00 [18.00, 30.00] | 28.00 [27.00, 30.00] | 18.00 [15.00, 19.50] | <0.001 |
| Age (years) | 47.00 [40.00, 55.00] | 46.00 [39.00, 51.00] | 48.00 [43.00, 53.00] | 0.699 |
| Males, n (%) | 13 (43.33) | 7 (46.67) | 6 (40.00) | 1.000 |
| Course (years) | 13.50 [10.00, 18.00] | 13.00 [9.00, 18.00] | 14.00 [10.00, 22.00] | 0.371 |
| Junior high school or above, n (%) | 12 (40.00) | 7 (46.67) | 5 (33.33) | 0.710 |
| TC (mmol/L) | 4.50 [4.00, 5.10] | 4.60 [4.00, 5.20] | 4.40 [3.80, 5.00] | 0.575 |
| LDL-C (mmol/L) | 2.78 [2.19, 3.16] | 2.78 [2.25, 3.27] | 2.78 [2.17, 3.16] | 0.868 |
| HDL-C (mmol/L) | 0.92 [0.82, 1.13] | 1.00 [0.80, 1.19] | 0.90 [0.82, 1.05] | 0.468 |
| NHDL-C (mmol/L) | 3.59 [2.85, 3.99] | 3.63 [2.98, 4.15] | 3.57 [2.83, 3.99] | 0.678 |
| TG (mmol/L) | 1.60 [1.00, 2.20] | 1.60 [0.70, 2.40] | 1.60 [1.00, 1.90] | 0.983 |
| ApoAI (g/L) | 1.32 [1.25, 1.52] | 1.37 [1.21, 1.55] | 1.31 [1.25, 1.51] | 0.493 |
| ApoB (g/L) | 0.99 [0.82, 1.14] | 1.00 [0.79, 1.14] | 0.97 [0.84, 1.18] | 0.836 |
| FBG (mmol/L) | 4.52 [4.17, 5.07] | 4.47 [4.27, 5.07] | 4.56 [3.96, 5.19] | 0.694 |
| CRP (mg/L) | 5.00 [3.00, 7.00] | 4.00 [3.00, 7.00] | 6.00 [2.00, 8.00] | 0.531 |
| BMI (kg/m^2^) | 22.14 [20.75, 23.74] | 21.36 [20.75, 22.41] | 22.51 [20.61, 25.93] | 0.263 |
| Hypertension, n (%) | 5 (16.67) | 2 (13.33) | 3 (20.00) | 1.000 |
| Diabetes, n (%) | 4 (13.33) | 2 (13.33) | 2 (13.33) | 1.000 |
| Clozapine, n (%) | 7 (23.33) | 3 (20.00) | 4 (26.67) | 1.000 |
| Olanzapine, n (%) | 2 (6.67) | 2 (13.33) | 0 | 0.483 |
| Risperidone, n (%) | 14 (46.67) | 9 (60.00) | 5 (33.33) | 0.272 |
| Quetiapine, n (%) | 2 (6.67) | 2 (6.67) | 2 (6.67) | 1.000 |

Quantitative variables: median [IQR]; qualitative variables: frequency (%), and analyzed by Fisher’s exact test due to the sample size < 40.

Abbreviations: CN, cognitive normal; CI, cognitive impairment; TC, total cholesterol; LDL-C, low density lipoprotein cholesterol; HDL-C, high density lipoprotein cholesterol; NHDL-C, non-high density lipoprotein cholesterol; TG, triglyceride; ApoA1, apolipoprotein A1; ApoB, apolipoprotein B; FBG, fasting blood glucose; CRP, C-reaction protein; BMI, body mass index.

**Supplementary Table 4** Differential metabolites between cognitive impairment and cognitive normal in patients with schizophrenia

| **MS2 name** | **rt** | **mz** | **Ion** | **Super Class** | **MEAN CI** | **MEAN NC** | **Change**  **(CI *vs* NC)** | **VIP** | **P-VALUE** | **FOLD CHANGE** |
| --- | --- | --- | --- | --- | --- | --- | --- | --- | --- | --- |
| N-(5-Methyl-3-oxohexyl)alanine | 378.43 | 202.1434 | [M+H]1+ | Organic acids and derivatives | 0.009 | 0.004 | Up | 2.214 | 0.032 | 2.601 |
| 1-Acetoxy-2-hydroxy-5,12,15-heneicosatrien-4-one | 173.20 | 379.2830 | [M+H]1+ | Lipids and lipid-like molecules | 0.004 | 0.002 | Up | 1.837 | 0.032 | 1.858 |
| (3beta,5alpha,6beta,9alpha,22E,24R)-5,9-Epidioxyergosta-7,22-diene-3,6-diol | 103.78 | 445.3303 | [M+H]1+ | Lipids and lipid-like molecules | 0.004 | 0.002 | Up | 1.752 | 0.025 | 1.475 |
| Theaspirone A | 267.32 | 209.1533 | [M+H]1+ | Organic oxygen compounds | 0.014 | 0.015 | Down | 1.827 | 0.010 | 0.901 |
| gamma-Glutamylleucine | 372.31 | 261.1438 | [M+H]1+ | Organic acids and derivatives | 0.006 | 0.008 | Down | 1.883 | 0.030 | 0.762 |
| 1-Arachidonoylglycerophosphoinositol | 268.67 | 621.3005 | [M+H]1+ | Lipids and lipid-like molecules | 0.008 | 0.011 | Down | 1.906 | 0.018 | 0.785 |
| 1,7-Dimethylguanosine | 206.62 | 312.1287 | [M+H]1+ | Nucleosides, nucleotides, and analogues | 0.011 | 0.014 | Down | 1.922 | 0.020 | 0.814 |
| 1,2,3,4-Tetrahydro-b-carboline-1,3-dicarboxylic acid | 312.21 | 261.0873 | [M+H]1+ | Alkaloids and derivatives | 0.018 | 0.021 | Down | 1.771 | 0.040 | 0.871 |
| Hexadecanedioic acid mono-L-carnitine ester | 292.70 | 430.3154 | [M+H]1+ | Lipids and lipid-like molecules | 0.007 | 0.010 | Down | 1.933 | 0.034 | 0.645 |
| Pyro-L-glutaminyl-L-glutamine | 183.15 | 258.1077 | [M+H]1+ | Organic acids and derivatives | 0.017 | 0.021 | Down | 1.838 | 0.048 | 0.819 |
| 7-Methylinosine | 149.25 | 283.1026 | [M+H]1+ | Nucleosides, nucleotides, and analogues | 0.012 | 0.016 | Down | 2.330 | 0.010 | 0.754 |
| 1-Pyrroline | 406.08 | 70.0656 | [M+H]1+ | Organoheterocyclic compounds | 0.035 | 0.041 | Down | 1.357 | 0.038 | 0.864 |
| trans-S-(1-Propenyl)-L-cysteine | 203.96 | 162.0579 | [M+H]1+ | Organic acids and derivatives | 0.016 | 0.022 | Down | 1.918 | 0.040 | 0.717 |
| N-Methylhistamine | 233.00 | 126.1024 | [M+H]1+ | Organic nitrogen compounds | 0.058 | 0.065 | Down | 1.787 | 0.040 | 0.892 |
| Dopamine 3-O-sulfate | 146.59 | 234.0426 | [M+H]1+ | Organic acids and derivatives | 0.007 | 0.014 | Down | 2.701 | 0.000 | 0.466 |
| 2-Pyrrolidinone | 468.66 | 86.0604 | [M+H]1+ | Organoheterocyclic compounds | 0.059 | 0.070 | Down | 1.668 | 0.035 | 0.844 |
| 3-Amino-2-piperidone | 209.28 | 115.0866 | [M+H]1+ | Organic acids and derivatives | 0.055 | 0.068 | Down | 1.395 | 0.045 | 0.804 |
| Sphinganine | 117.66 | 302.3041 | [M+H]1+ | Organic nitrogen compounds | 0.037 | 0.051 | Down | 1.715 | 0.034 | 0.736 |
| 1-Methylhypoxanthine | 149.30 | 151.0610 | [M+H]1+ | Organoheterocyclic compounds | 0.065 | 0.085 | Down | 2.524 | 0.003 | 0.764 |
| N6-Methyladenosine | 295.63 | 282.1184 | [M+H]1+ | Nucleosides, nucleotides, and analogues | 0.129 | 0.152 | Down | 2.015 | 0.019 | 0.850 |
| 3-Dehydrosphinganine | 71.39 | 300.2883 | [M+H]1+ | Organic oxygen compounds | 0.059 | 0.083 | Down | 2.236 | 0.019 | 0.716 |
| Oleamide | 71.39 | 282.2784 | [M+H]1+ | Lipids and lipid-like molecules | 0.088 | 0.123 | Down | 2.280 | 0.016 | 0.714 |
| 3-Methylguanine | 199.37 | 166.0721 | [M+H]1+ | Organoheterocyclic compounds | 0.197 | 0.236 | Down | 2.138 | 0.010 | 0.835 |
| N5-Carboxyaminoimidazole ribonucleotide | 321.23 | 340.0550 | [M+H]1+ | Organic oxygen compounds | 0.286 | 0.340 | Down | 2.026 | 0.043 | 0.842 |
| PC(20:3(5Z,8Z,11Z)/P-18:0) | 37.16 | 796.6181 | [M+H]1+ | Lipids and lipid-like molecules | 0.447 | 0.505 | Down | 1.901 | 0.041 | 0.885 |
| 2-[(Methylthio)methyl]-2-butenal | 388.77 | 131.0531 | [M+H]1+ | Organic oxygen compounds | 0.636 | 0.710 | Down | 1.862 | 0.024 | 0.895 |
| 5-Ethyl-2,4-dimethyloxazole | 66.97 | 126.0911 | [M+H]1+ | Organoheterocyclic compounds | 0.192 | 0.281 | Down | 2.293 | 0.007 | 0.682 |
| Acetone cyanohydrin | 100.71 | 86.0603 | [M+H]1+ | Organic oxygen compounds | 0.279 | 0.369 | Down | 1.474 | 0.025 | 0.756 |
| Isoleucyl-Alanine | 370.43 | 203.1384 | [M+H]1+ | Organic acids and derivatives | 0.403 | 0.532 | Down | 1.351 | 0.027 | 0.758 |
| Asymmetric dimethylarginine | 496.77 | 203.1500 | [M+H]1+ | Organic acids and derivatives | 2.496 | 2.885 | Down | 1.716 | 0.034 | 0.865 |
| Propionylcarnitine | 296.43 | 218.1382 | [M+H]1+ | Lipids and lipid-like molecules | 1.401 | 2.222 | Down | 2.304 | 0.004 | 0.630 |
| L-Isoleucine | 291.66 | 132.1017 | [M+H]1+ | Organic acids and derivatives | 6.038 | 7.262 | Down | 2.040 | 0.010 | 0.831 |
| Pyrrolidonecarboxylic acid | 388.76 | 130.0497 | [M+H]1+ | Organic acids and derivatives | 11.106 | 12.500 | Down | 1.926 | 0.018 | 0.888 |
| D-Glutamine | 388.76 | 147.0759 | [M+H]1+ | Organic acids and derivatives | 17.899 | 20.110 | Down | 1.966 | 0.013 | 0.890 |
| Creatine | 364.08 | 132.0764 | [M+H]1+ | Organic acids and derivatives | 5.155 | 7.418 | Down | 1.166 | 0.009 | 0.695 |
| Choline | 274.32 | 104.1071 | [M+H]1+ | Organic nitrogen compounds | 23.231 | 26.047 | Down | 1.618 | 0.016 | 0.892 |
| D-Proline | 326.66 | 116.0705 | [M+H]1+ | Organic acids and derivatives | 13.790 | 18.102 | Down | 2.058 | 0.005 | 0.762 |

rt, retention time (s); m/z, mass to charge ratio; VIP: variable importance in the projection.

**Supplemental Table 5** Enriched pathways and related differential metabolites

| **Pathway** | **Total** | **Hits** | **%** | **Raw *P*** | **-ln(*P*)** | **Impact** | **Metabolites of hits** |
| --- | --- | --- | --- | --- | --- | --- | --- |
| D-Glutamine and D-glutamate metabolism | 11 | 2 | 0.18 | 0.002* | 6.391 | 0.171 | D-Glutamine; Pyrrolidonecarboxylic acid |
| Sphingolipid metabolism | 25 | 2 | 0.08 | 0.009* | 4.741 | 0.205 | Sphinganine; 3-Dehydrosphinganine |
| Glycine, serine and threonine metabolism | 48 | 2 | 0.04 | 0.030* | 3.493 | 0.001 | Choline; Creatine |
| Arginine and proline metabolism | 77 | 2 | 0.03 | 0.072 | 2.635 | 0.045 | D-Proline; Creatine |
| Valine, leucine and isoleucine biosynthesis | 27 | 1 | 0.04 | 0.146 | 1.921 | 0.013 | L-Isoleucine |
| Glycerophospholipid metabolism | 39 | 1 | 0.03 | 0.205 | 1.585 | 0.021 | Choline |
| Valine, leucine and isoleucine degradation | 40 | 1 | 0.03 | 0.210 | 1.562 | 0.000 | L-Isoleucine |
| Aminoacyl-tRNA biosynthesis | 75 | 1 | 0.01 | 0.359 | 1.025 | 0.000 | L-Isoleucine |

Total, number of all metabolites in this pathway; Hits, number of differential metabolites hit this pathway; %, percentage of differential metabolites in this pathway; Metabolites of hits, differential metabolites hit this pathway.
